# The Abrogation of Phosphorylation Plays a Relevant Role in the CCR5 Signalosome Formation with Natural Antibodies to CCR5

**DOI:** 10.3390/v10010009

**Published:** 2017-12-28

**Authors:** Assunta Venuti, Claudia Pastori, Gabriel Siracusano, Rosamaria Pennisi, Agostino Riva, Massimo Tommasino, Maria Teresa Sciortino, Lucia Lopalco

**Affiliations:** 1 Division of Immunology, Transplantation and Infectious Diseases, DIBIT-San Raffaele Scientific Institute, 20132 Milan, Italy; pastori.claudia@hsr.it (C.P.); siracusano.gabriel@hsr.it (G.S.); lopalco.lucia@hsr.it (L.L.); 2 Infections and Cancer Biology Group, International Agency for Research on Cancer, 150 Cours Albert Thomas, 69372 Lyon CEDEX 08, France; tommasinom@iarc.fr; 3 Department of Chemical Biological Pharmaceutical and Environmental Sciences, University of Messina, 98166 Messina, Italy; rpennisi@unime.it (R.P.); mtsciortino@unime.it (M.T.S.); 4 Third Division of Infectious Diseases, Luigi Sacco Hospital, University of Milan, 20157 Milan, Italy; agostino.riva@unimi.it

**Keywords:** CCR5, anti CCR5 antibodies, CCR5 signalosome, HIV infection, HIV protection, CCR5 based therapy, ESN, LTNP

## Abstract

The exposure to CCR5 (CC chemokine receptor 5) specific natural antibodies in vitro produces a Class B β-arrestin2-dependent CCR5 retention with the aid of ERK1, due to the formation of a CCR5 signalosome, which remains stable for at least 48 h. Considering that β-arrestins and MAPKs are receptive to environmental signals, their signal complexes could be one of the key junction for GPCRs internalization related signal transduction. Here, we demonstrate that, in T cells, the phosphorylation status of either CCR5 receptor or ERK1 protein is necessary to drive the internalized receptor into the early endosomes, forming the CCR5 signalosome. In particular, our data show that β-arrestin2/ERK1 complex is a relevant transducer in the CCR5 signaling pathway. Understanding the mechanism of CCR5 regulation is essential for many inflammatory disorders, tumorigenesis and viral infection such as HIV.

## 1. Introduction

Chemokine receptors belong to seven-transmembrane proteins (7TMRs, also called G-protein coupled receptors, GPCRs), which play a fundamental role in a multiplicity of developmental and chemotactic events [1,2].

After stimulation, these receptors activate a restricted number of effectors (e.g., adenylate cyclase and phospholipase C), which consequently influence the intracellular concentrations of second messengers (e.g., inositol 1,4,5 trisphosphate, cyclic AMP, diacylglycerol, and Ca^2+^) and their relative target proteins, including protein kinase A (PKA), protein kinase C (PKC) and the related substrates. This in turn results in regulation of several cellular functions, via heterotrimeric G proteins, and also G protein-dependent and -independent activation of mitogen activated protein kinases (MAPKs), including the extracellular signal-regulated kinase (ERK) cascade [3]. Recent works indicate that β-arrestins, normally involved in 7TMRs desensitization and internalization, might also interact with non-receptor kinases, such as ERK, to form complexes for signal propagation and transduction [1,2,3,4,5]. Indeed, it has been established that β-arrestins can act as a scaffold to form a complex containing the receptor and the effectors [2]; in fact, the 7TMRs could be divided into two subgroups depending on the stability of interaction with β-arrestins after agonist binding [4,6].

Human CC chemokine receptor 5 (CCR5), a member of the superfamily of 7TMRs, has been identified as the receptor for chemokines such as MIP-1α (Macrophage inflammatory protein 1α, also CCL3), MIP-1β (Macrophage inflammatory protein-1β, also CCL4), RANTES (Regulated on Activation, Normal T Cell Expressed and Secreted, also CCL5) [7], and also serves as a coreceptor for the entry of R5-HIV-1 strains into the cells, in association with CD4 [8].

Natural antibodies, directed to the first external loop of CCR5 (ECL1), have been found in HIV infected Long Term Non-Progressor subjects (LTNPs) and are able to induce a long lasting internalization (48 h) of the receptor, which inhibits HIV entry [9]. Even more recently, our group has reported that, in T cells, natural CCR5 antibodies induce a CCR5-negative phenotype with downstream enrollment of β-arrestin2, which results in the formation of a stable CCR5 signalosome with the internalized CCR5, β-arrestin2 and ERK1 proteins [10].

As β-arrestins and MAPKs are receptive to environmental signals, their signal complex could be one of the key regulatory points of signal transduction related to GPCR internalization. Our data demonstrate that the phosphorylation status of the major players of the CCR5 signalosome, in the cellular environment, is responsible for a stable CCR5 sequestration.

## 2. Materials and Methods

### 2.1. Cell Lines

R5-SupT1-transducted cell lines, engineered and kindly provided by H. Garg, are CCR5-expressing cell lines mentioned as SupT1-R5 clones L23 (expressing low level of CCR5 at cell surface) and M10 (expressing medium level of CCR5 at cell surface) as previously reported [10]. Briefly, M10 cell line were used to perform the IP assay considering that the higher density of CCR5 on cell membrane and L23 were used to induce downregulation under physiological condition, thus mimicking the condition with primary CD4^+^ T cells either in vitro (as we already demonstrated in our previous work [11]) or ex vivo in subjects positive for anti-CCR5 antibodies (as reported in [9]). The cells were cultured at 37 °C in a 5% CO_2_ incubator and were propagated in RPMI 1640 (Lonza, Verviers, Belgium) supplemented with 10% FCS (Lonza), 2 mM l-glutamine, 100 U/mL penicillin, 100 U/mL streptomycin and with 3 μg/mL of blasticidin (Calbiochem, Darmstadt, Germany).

### 2.2. Reagents

Anti-p-CKR-5(E11/19), which specifically recognizes phosphoserine at position 349 (a specific GRK phosphorylation site) [12], anti-CKR-5 (D6), anti-GAPDH MAbs, anti-p-ERK1/2 (Thr 202/Tyr 204), anti-ERK1 (C-16), and Rab5 (FL-215) polyclonal antibodies were obtained from Santa Cruz Biotechnology (Santa Cruz, CA, USA). β-Arrestin1/2 (D24H9) rabbit monoclonal antibody, which detects endogenous level of total β-arrestin1 and -2 proteins, shows a single band at 50 KDa, as reported in the datasheet, was purchased from Cell Signaling Technology (Beverly, MA, USA). Secondary HRP-conjugated goat anti-rabbit IgG and goat anti-mouse IgG were purchased from Millipore; secondary FITC-conjugated goat anti-mouse IgG was from ICN Biomedicals (Aurora, OH, USA). Secondary HRP-conjugated anti-rabbit and anti-mouse IgG VeriBlot for IP were from Abcam (Cambridge, UK).

### 2.3. Study Population

The pool of serum samples from 5 LTNPs positive for anti-CCR5 natural antibodies (CCR5 Ab Pos) included in this study (No. 4, No. 11, No. 20, No. 21 and No. 22) have been previously characterized and described [9]. Briefly, they had documented seropositivity of ≥7 years, presented no HIV-related symptoms during this period, had never received any antiretroviral treatment, were in healthy condition, and had their CD4^+^ T-cell count persistently higher than 500 cells/μL of blood (mean 725 cells/μL). The group included 2 women and 3 men ranging in age between 35 and 52 years. The serum samples from these subjects contain antibodies that specifically recognized ECL1 of CCR5, induced complete receptor internalization after 48 h of incubation to either primary CD4^+^ T cells or T cell lines, blocked HIV infection and transcytosis, which mimics mucosal transmission [13]. A pool of serum samples from LTNPs negative for anti-CCR5 antibodies (CCR5 Ab Neg), which included 3 women and 2 men, ranging in age between 33 and 48, was used as negative control, as previously described [9,11].

### 2.4. Ethics Statement

The investigations were approved by the Institutional review board named “Comitato Etico della Fondazione San Raffaele del Monte Tabor”, Milan, Italy, (May 2002). A written informed consent was signed by all subjects and all methods were performed in accordance with the relevant Italian guidelines and regulations.

### 2.5. CCR5 Internalization Assay

The internalization assay was performed accordingly with our experimental conditions previously published [10,12]. Briefly, R5-SupT1-L23 or M10 cells were exposed to CCR5 Ab Pos (1/30), and to 2 μg/mL RANTES (R&D Systems, MN, USA) as a positive control, at 37 °C for 30 min. The cells were then washed and incubated for an additional 120 min [10,12]. CCR5 Ab Neg was used as a negative control [10,12]. When indicated, the cells were pre-treated for 1 h with staurosporine (a pan-kinases inhibitor) (50 nM) (Sigma Aldrich, St. Louis, MO, USA). As staurosporine has been reconstituted in DMSO, the same percentage of DMSO diluted in RPMI was used as a negative control (unpublished data). The detection of cytoplasmic CCR5 was obtained by using immunofluorescence and analyzed on a Leica DMRE fluorescence microscope, according to Venuti et al., 2016 [10].

### 2.6. Western Blotting

The cells were lysed in RIPA lysis buffer (50 mM Tris-HCl, pH 7.5, 10 mM MgCl_2_, 150 mM NaCl, 0.5% sodium deoxycholate, 1% Nonidet P-40) in the presence of complete protease inhibitor cocktail (Roche, Indianapolis, IN, USA). Samples were resolved by SDS–PAGE and transferred to nitrocellulose membranes (Amersham Pharmacia Biotech AB, Uppsala, Sweden). After blocking in 5% non-fat milk, the membranes were incubated overnight at 4 °C with the appropriate primary antibody and then probed, at room temperature for 1 h, with HRP-tagged secondary antibodies. The chemiluminescent signals were detected by ECL method (Millipore, Burlington, MA, USA). Comparative analysis of the bands was performed by quantitative densitometry and concomitant use of the TINA software (version 2.10, Raytest, Straubenhardt, Germany). The normalization was done per each band based on the density of GAPDH signal per each line.

### 2.7. Immunoprecipitation

The immunoprecipitation assay was carried out as previously described [10,11,14]. Briefly, the cells were treated with CCR5 Ab Pos and relative controls, then were collected, washed twice in PBS and lysed with cold lysis buffer (20 mM Tris-HCl, pH 8, 1 mM EDTA, 200 mM NaCl, 1% Nonidet P-40, 2 mM DTT, 0.1 mM Na_3_VO_4_, 10 mM NaF, 0.1 μg/mL Protease Inhibitors). The protein samples were precleared with 50% of protein-A slurry for 16 h. Immunoprecipitations were performed with the indicated antibodies pre-adsorbed on protein A-Sepharose beads (Amersham Pharmacia Biotech AB) for 2 h at 4 °C [10,11,14]. In particular, the monoclonal antibody anti-CKR-5 (D6) recognizes ECL2 and ECL3 of CCR5, thus it does not interfere with the activity of CCR5 Ab Pos, which are specific for ECL1 domain of CCR5 only (unpublished data). After overnight incubation with the protein extracts, complexate-beads were resolved by SDS–PAGE and transferred to nitrocellulose membranes (Amersham Pharmacia Biotech AB, Uppsala, Sweden). Immunoblotting was performed with β-arrestin1/2, ERK1 and Rab5 antibodies and immunoreactivity was revealed by using secondary antibodies specific for IP (Abcam). The chemiluminescent signals were detected by ECL method (Millipore).

### 2.8. siRNA Nucleofection

Chemically synthesized siRNAs sequences (Eurofins Biolab Srl, Vimodrome, Italy) specific for human β-arrestin1 and -2 and GAPDH (NSsiRNA) were nucleofected using an Amaxa Nucleofector Device (Lonza) in accordance with the manufacturer’s instructions. The cells were then seeded onto 24 multi-well plates for 5 h and treated in accordance with the experimental procedure described in our previous works [10,11,14].

### 2.9. Statistical Analysis

**Figure 1:**
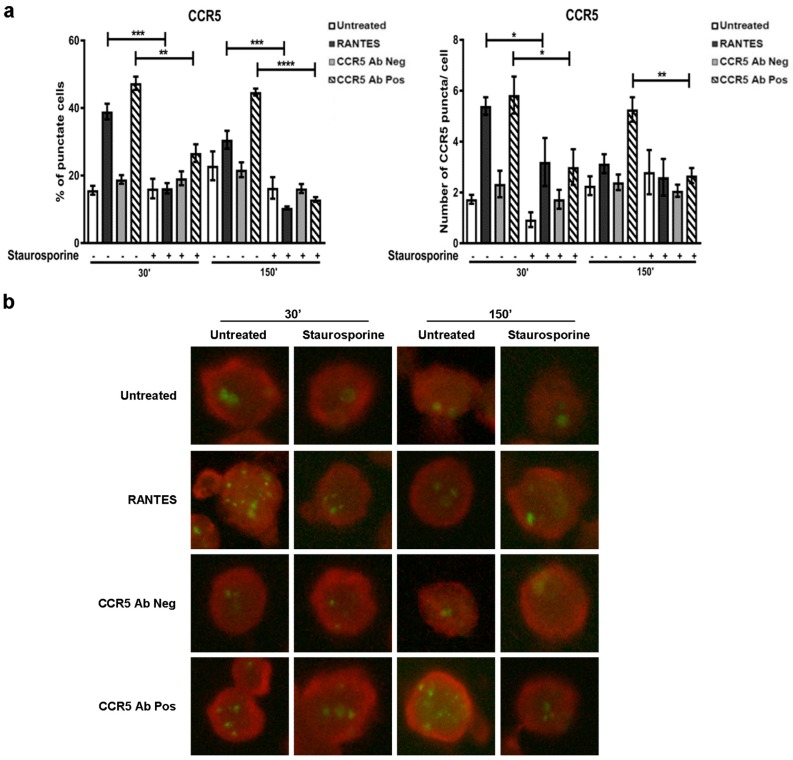
CCR5 internalization in the presence of a pan-kinase inhibitor (staurosporine). After 1 h of pre-treated with staurosporine (50 nM), R5-SupT1-L23 cells were stimulated, or not, with CCR5 Ab Pos, RANTES (positive control) and CCR5 Ab Neg (negative control). The cells were harvested at 30 min and at 150 min (30 min incubation, wash, additional 120 min incubation in medium without stimuli). (**a**) The percentage of cells with the punctate form of CCR5 (left) and the number of CCR5 puncta per cell (right) in cells treated or not with the stimuli, with or without staurosporine treatment, stained with anti-CKR5(D6), are stated. Data are representative of three independent experiments. Bar graphs represented mean ± standard deviation (SD) of three independent experiments. Student’s *t*-test was performed and *p*-values are shown. * *p* ≤ 0.05, ** *p* ≤ 0.01, *** *p* ≤ 0.001, **** *p* ≤ 0.0001; (**b**) a representative immunofluorescence image of cells positive for CCR5 detection (lens magnification: 63×). Evans Blue dye was used as a counter stain.

**Figure 2:**
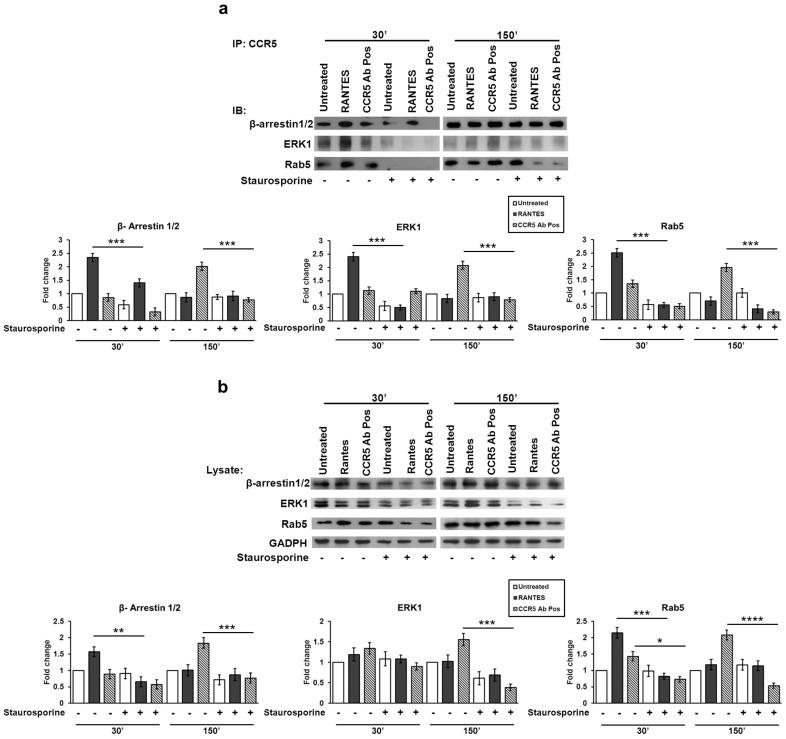
Role of the phosphorylation status in the CCR5 signalosome formation. After 1 h of pre-treated with staurosporine (50 nM), R5-SupT1-M10 cells were stimulated, or not, with CCR5 Ab Pos, RANTES (positive control). The cells were harvested at 30 min and at 150 min (30 min incubation, wash, additional 120 min incubation in medium without stimuli). (**a**) Co-IP on cell lysates was performed for CCR5 followed by immunoblots for β-arrestin1/2, ERK1 and Rab5 expression; (**b**) western blot for β-arrestin1/2, ERK1 and Rab5 in total cell lysates was accomplished. Band density was determined with the TINA software (version 2.10, Raytest, Straubenhardt, Germany), and it is shown as fold change over a housekeeping gene. Bar graphs represented mean ± SD of three independent experiments. Student’s *t*-test was performed and *p*-values are shown. * *p* ≤ 0.05, ** *p* ≤ 0.01, *** *p* ≤ 0.001, **** *p* ≤ 0.0001. Data are representative of three independent experiments.

**Figure 3:**
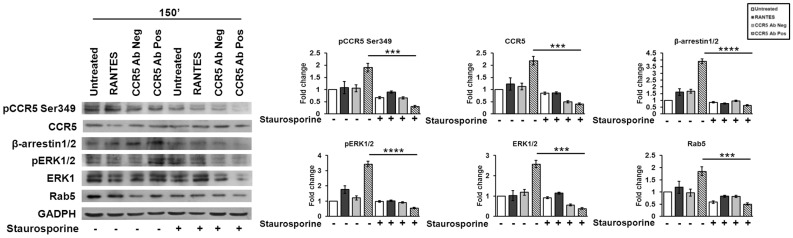
Contribution of phosphorylation activity in the CCR5 regulation. After 1 h of pre-treated with staurosporine (50 nM), R5-SupT1-M10 cells were stimulated, or not, with CCR5 Ab Pos, RANTES (positive control) and CCR5 Ab Neg (negative control). The cells were harvested at 150 min (30 min incubation, wash, additional 120 min incubation in medium without stimuli). Western blot analysis from total extracts were performed to evaluate phosphorylated and non-phosphorylated forms of CCR5 and ERK1, β-arrestin1/2, and Rab5. Band density was determined with the TINA software (version 2.10, Raytest, Straubenhardt, Germany) and it is shown as fold change over a housekeeping gene. Bar graphs represented mean ± SD of three independent experiments. Student’s *t*-test was performed and *p*-values are shown. *** *p* ≤ 0.001, **** *p* ≤ 0.0001. Data are representative of three independent experiments.

The two-tailed Student’s *t*-test was used and Prism version 5.0a (GraphPad Software, La Jolla, CA, USA) was used to analyze the data. Figure 1, Figure 2 and Figure 3 show *p*-values: * stands for *p* ≤ 0.05, ** stands for *p* ≤ 0.01, *** stands for *p* ≤ 0.001 and **** stands for *p* ≤ 0.0001.

## 3. Results

### 3.1. Staurosporine Treatment Reduces the Cytoplasmic Accumulation of CCR5

It is well demonstrated that natural antibodies to CCR5 receptor (CCR5 Ab Pos), detected in the sera of LTNPs, induce a long-lasting internalization (48 h) with the recruitment of β-arrestin2 and ERK1 proteins [9,10,11]. Activation of seven transmembrane receptors (7TMRs) promotes the formation of stable complexes between activated 7TMRs, activated and ubiquitinated β-arrestin and phosphorylated ERK named “signalosome” [10].

Taking into account that cellular signaling pathways trigger protein–protein interactions mediated by phosphorylation and dephosphorylation events [15], here we wondered if the inhibition of specific kinases activity may affect the CCR5 internalization mediated by the agonist-induced β-arrestin-ERK interaction.

The experiments were carried out in the R5-SupT1-L23 T lymphoblastoid cell line that shows an activation status and a CCR5 cell surface level comparable to human CD4^+^ T lymphocytes, as previously reported [11]. We used a pool of five LTNPs sera, each containing CCR5 antibodies (CCR5 Ab Pos), or not (CCR5 Ab Neg, used as a negative control), as previously described and characterized by our group [9,10,11]; as a positive control, the cells were also stimulated with the chemokine RANTES [11].

To this end, cells were pre-treated with 50 nM staurosporine, a broad spectrum protein kinases inhibitor, and then stimulated for 30 min with CCR5 Ab Pos, CCR5 Ab Neg and with RANTES or not. The first group of samples was collected after 30 min of stimulation and it is used as a control of the classical short term kinetics of CCR5 internalization (30 min) described for its natural ligands, such as RANTES [7,16,17,18,19]. After 30 min, the second group of samples was washed and incubated for additional 120 min (i.e., total incubation time: 150 min): this time was chosen considering that the stimulation with CCR5 Ab Pos induces a CCR5 downregulation starting at 150 min only, as previously published [9,10,11].

To evaluate the CCR5 localization at single cell level, we performed an immunofluorescence analysis by using a specific antibody, which binds the external regions of CCR5 (amino acids 66–250).

At 30 min, the percentage of CCR5 positive cells and the number of specific CCR5 puncta per cell treated with RANTES and with CCR5 Ab Pos showed a statistically significant increase (*p* ≤ 0.001) in comparison with the respective controls. As expected, the pre-treatment with staurosporine statistically significantly reduced the percentage of punctuate cells (*p* ≤ 0.001 for RANTES and *p* ≤ 0.01 for CCR5 Ab Pos) and the number of CCR5 puncta per cells (*p* ≤ 0.01) upon stimulation with both ligands tested (Figure 1).

Conversely, at 150 min, the stimulation with CCR5 Ab Pos, but not with RANTES, substantially increased (*p* ≤ 0.001) the percentage of cytoplasmic CCR5 puncta structures, as expected. In addition, the presence of staurosporine still determined a reduction both in the percentage of CCR5 positive cells (*p* ≤ 0.0001) and in the number of positive specific CCR5 puncta per cell (*p* ≤ 0.01), in the cells stimulated with CCR5 Ab Pos (Figure 1).

In addition, at this time point, the presence of staurosporine reduced also the basal percentage of CCR5 positive cells (*p* ≤ 0.001) in the cells stimulated with RANTES (Figure 1).

These observations indicate that the phosphorylation status of the proteins involved in the complex (CCR5, ERK1 and β-arrestin) is relevant for the cytoplasmic accumulation of CCR5 as shown by the reduction in the CCR5 retention in all the samples treated with staurosporine.

### 3.2. Protein Kinases Activity Plays a Key Role for the CCR5 Signalosome Formation and Stabilization

To verify if the inhibition of protein kinases activity could be a restriction factor for the CCR5 signalosome formation, we analyzed the early temporal association of the internalized receptor CCR5 with the endogenous ERK1 and β-arrestin1/2 by immunoprecipitation assay, after pre-treatment or not with staurosporine, upon stimulation with RANTES and CCR5 Ab Pos, as described above. As agonist binding to CCR5 triggers the clustering of the receptor in clathrin-coated pits, which are delivered to the early endosomes [11,19], we also evaluated the interactions with Rab5 protein, a regulatory factor in the early endocytic pathway [4].

The experiment was performed in R5-SupT1 M10 cell line that has higher levels of CCR5 expression than R5-SupT1 L23 [10]. After 30 min and 150 min of stimulation, the cells were collected, lysed and adsorbed with CCR5-antibody-beads, as described in Materials and Methods. Western blotting analysis showed that β-arrestin1/2, ERK1 and Rab5 proteins are able to interact with CCR5.

In detail, Figure 2a shows a specific induction of the three proteins evaluated after stimulation with RANTES and CCR5 Ab Pos, at 30 min and 150 min respectively.

Interestingly, at 30 min, the staurosporine treatment, which inhibits protein kinases activity, strongly reduced the basal level interaction of β-arrestin1/2 and ERK1 with CCR5; its presence also statistically significantly inhibited their interaction after RANTES stimulation (*p* ≤ 0.0001), which is notable to induce the signalosome formation at this time point, and after CCR5 Ab Pos stimulation as well.

In addition, staurosporine treatment completely abrogated the interaction of CCR5 with Rab5 in all the tested conditions.

As expected, the immunoblot at 150 min highlighted a statistically significant decrease in the complex formation in the presence of staurosporine inhibitor upon stimulation with CCR5 Ab Pos only; this is in line with our previous results showing that, at this time point, RANTES was not able to induce the CCR5 signalosome [10].

A parallel Western blot analysis of the lysates was done to prove that the staurosporine treatment can also affect the accumulation of the proteins involved in the CCR5 signalosome complex. As shown in Figure 2b, the staurosporine treatment statistically significantly reduced the retention of β-arrestin1/2 and ERK1 (*p* ≤ 0.001) in the cells incubated with CCR5 Ab Pos compared to the non-treated control ones, at 150 min. Furthermore, the inhibition of protein kinases activity statistically significantly affected the accumulation of Rab5 protein, both at 30 min (*p* ≤ 0.01) and 150 min (*p* ≤ 0.0001), upon stimulation with CCR5 Ab Pos. As expected, the cells stimulated with RANTES and treated with staurosporine showed a statistically significant decrease of β-arrestin1/2 (*p* ≤ 0.01) and Rab5 (*p* ≤ 0.001) at 30 min only.

These findings suggest that, after internalization, the CCR5 complex is driven to early endosomes directly but the inhibition of protein kinases cascade deeply alters the protein–protein interaction among the CCR5 signalosome in a ligand-independent manner.

### 3.3. Staurosporine Treatment Alters the Regulation of the Core Components of CCR5 Pathway

It has been published that the binding of natural ligands to CCR5 induces the phosphorylation of four distinct C-terminal serine residues mediated by GPCR kinase (GRK) and protein kinase C (PKC) [17]. Based on studies of phosphorylation events in vivo, Pollok-Kopp and colleagues have produced monoclonal antibodies that specifically recognize either phosphorylated or non-phosphorylated CCR5; the phosphosite-specific antibodies showed that under ligand stimulation of the receptor, Serine 337 is exclusively phosphorylated by a PKC-mediated mechanism, while GRKs phosphorylate Serine 349 [12].

Considering that CCR5 Ab Pos induce the CCR5 signalosome formation starting at 150 min, with the involvement of GRK-mediated phosphorylation and consequent interaction with β-arrestin1/2, we investigated the effects of staurosporine inhibition on the regulation of the core components of the CCR5 signaling pathway activation. Thus, after 1 h of treatment with staurosporine inhibitor, the R5-SupT1-L23 cells were incubated with CCR5 Ab Pos and relative controls and then harvested at 150 min (150 min: 30 min incubation, wash, further 120 min incubation).

The results demonstrated that CCR5 Ab Pos triggered the phosphorylation on Ser349 with consequent accumulation of CCR5 as well as an increase of phosphorylated and non-phosphorylated form of ERK1 and of β-arrestin1/2 and Rab5 respectively. Conversely, the inhibition of protein kinases mediated by staurosporine statistically significantly reduced phosphorylation of both CCR5 Ser349 (*p* ≤ 0.001) and ERK1 (*p* ≤ 0.0001), as well as the accumulation of the non-phosphorylated form of these proteins (*p* ≤ 0.001) in the cells incubated with CCR5 Ab Pos compared with the non treated controls. No activity was observed in cells either treated or untreated with CCR5 Ab Neg and RANTES respectively (Figure 3).

The cells exposed to CCR5 Ab Pos and pre-treated with the protein kinases inhibitor showed a strong reduction of β-arrestin1/2 (*p* ≤ 0.0001) and Rab5 (*p* ≤ 0.001) proteins as well (Figure 3).

These data, taken together with those obtained by immunoprecipitation assay, clearly demonstrated that CCR5 and ERK1 phosphorylation status plays a fundamental role for the formation of CCR5 signalosome within the early endosomes compartment.

### 3.4. β-arrestin2 Is Crucial for the CCR5 Signalosome Formation

We have recently demonstrated that CCR5 Ab Pos induce a CCR5-negative phenotype with the specific contribution of β-arrestin2 [10]. Here, we carried out the same experiment to better define the role of β-arrestin2 in the prompt recruitment of the receptor complex to the early endosomes formation.

Briefly, R5-SupT1 M10 were transiently nucleofected with siRNAs specific for β-arrestin1, β-arrestin2 or both and a relative control (NS), incubated with CCR5 Ab Pos and the relative controls and then harvested at 150 min, as described in our previous works [10,11,14].

**Figure 4:**
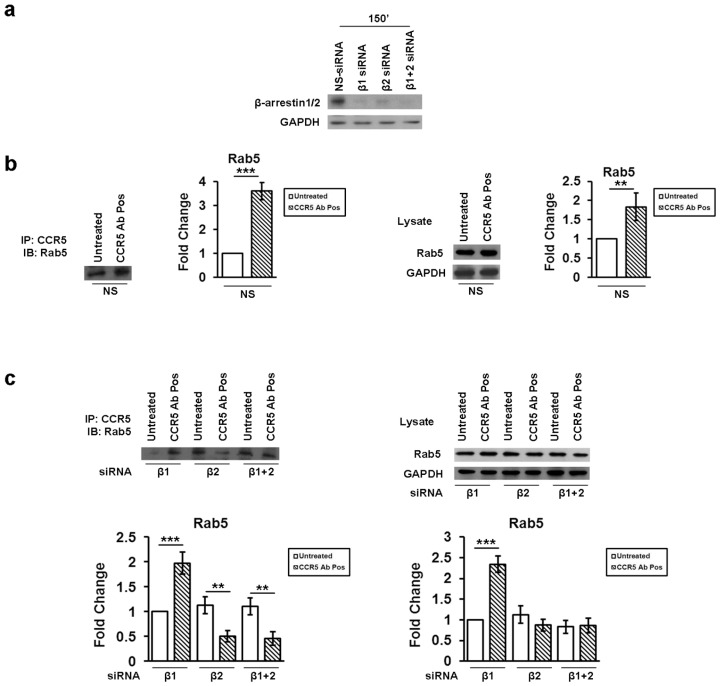
Role of siRNAs specific for β-arrestin1/2 in the interaction between CCR5 and Rab5. (**a**) Immunoblot analysis for β-arrestin1/2 and GAPDH in whole lysate from R5-SupT1-M10 cells nucleofected with siRNAs specific for β-arrestins: -1 (β1), -2 (β2) or both (β1+2) and with NS-siRNA (negative control); (**b,c**) after 5 h of nucleofection, the cells were collected at 150 min: 30 min incubation with CCR5 Ab Pos or not, wash, additional 120 min incubation in medium without stimuli. The immunoprecipitation of CCR5 with Rab5 was assessed by immunoblot. Total protein extracts were tested by Western blot as an experimental control. Band density was determined with the TINA software (version 2.10, Raytest, Straubenhardt, Germany) and it is shown as fold change over a housekeeping gene. Bar graphs represented mean ± SD of three independent experiments. Student’s *t*-test was performed and *p*-values are shown. ** *p* ≤ 0.01, *** *p* ≤ 0.001. Data are representative of three independent experiments.

The immunoblot showed that β-arrestin1/2 siRNAs duplexes specifically reduced protein expression levels (Figure 4a).

We performed an immunoprecipitation assay, in the cells nucleofected or not with siRNAs specific for β-arrestin1 and -2, to determine the possible interaction between the endogenous Rab5 protein and the internalized CCR5 receptor. At 150 min, Rab5 had a weak basal level interaction with the receptor and it was strongly engaged to CCR5 upon stimulation with CCR5 Ab Pos in the cells nucleofected with non-specific targeting siRNA (NS-siRNA) (Figure 4b). Moreover, the depletion of cellular level of β-arrestin1 did not affect the CCR5 Ab Pos-specific accumulation (Figure 4c); on the contrary, the silencing of β-arrestin2 or of both β-arrestin1 and -2, showed a decrease in the complex formation as shown by the weaker Rab5 binding (Figure 4c).

These data confirm that the CCR5 stimulation with CCR5 Ab Pos requires a β-arrestin2-dependent endocytosis and its depletion prevents the formation of the CCR5 signalosome into the early endosomes.

## 4. Discussion

Human CC chemokine receptor 5 (CCR5), a member of seven-transmembrane proteins (7TMRs, also called G protein-coupled receptors, GPCRs), is the main coreceptor for the entry of R5 tropic strain of HIV-1, in association with CD4 [8]. Binding of natural ligands induces a rapid phosphorylation of serine and threonine residues located in the C-terminal region of CCR5; this event promotes receptor internalization by the aid of nonvisual arrestins such as β-arrestin1 and β-arrestin2 [16,19,20].

Receptor interaction with β-arrestin triggers many conformational changes of the β-arrestin itself, which activate various kinases leading to specific signaling outcomes and physiological responses. The type of the phosphorylation barcode on cytoplasmic domains of GPCRs in association with the ubiquitination status of β-arrestin determines the stability of activated GPCR–β-arrestin complexes and their co-trafficking to endosomes [20].

On the other hand, β-arrestins are able to induce intracellular signal transduction mediated by protein–protein interactions with several proteins, including MAPKs [4,15,21,22,23,24,25]. Indeed, our group has recently demonstrated class B β-arrestin2-dependent CCR5 signalosome retention mediated by the exposure to natural antibodies to CCR5 [10].

The endocytic system recruits a very dynamic network of vesicles and endosomes, and the evolution of this network is important to understand the intracellular trafficking. Rab GTPases are specifically localized into various intracellular organelles [26,27,28]; in particular, Rab5 and Rab7 are mainly associated with early and late endosomes respectively, whereas Rab11 represents a marker of recycling endosomes [26,29,30,31]. As the phosphorylation and dephosphorylation events, in response to environmental stimuli, could regulate several protein interactions, our results demonstrate that the phosphorylation status of both CCR5 receptor and ERK1 protein is fundamental for addressing the internalized receptor into the early endosomes, forming the CCR5 signalosome. Further studies are necessary to better understand the CCR5 signalosome fate in endosomes network.

Antibodies (IgG and IgA) direct to ECL1 of CCR5 have been found in ESNs [32] as well as in LTNPs [33,34]. Interestingly, they could be generated independently of any exposure to foreign antigens or vaccines or elicited in the course of infectious diseases. Other natural Igs to CCR5 have also been found in healthy donors [35] and in CCR5 lacking subjects (Δ32 homozygous) after repeated sexual exposure to partner’s CCR5+ [36].

Anti ECL1-CCR5 antibodies induce a stable and long-lasting downregulation of CCR5 with the involvement of ERK1-based pathway [11], starting at 150 min and achieving the maximum level at 48 h [9,10,11]. This results in a block of HIV infection in CD4^+^ T lymphocytes [13], and this mechanism is different from that reported for all other CCR5 specific ligands [37], even though we cannot exclude that this mechanism is specific for T cells only.

The endocytic mechanism CCR5 Ab Pos mediated could be strictly related to their specific binding site, as natural CCR5 antibodies recognize a conformational epitope within the first extramembrane loop (ECL1) [9]. Conversely, the ligands bind the second external loop (ECL2) of CCR5; thus, the specific binding to each domain can differentially modulate the signaling, which could influence the receptor fate, resulting in a internalization pathway different from that one showed for the other common CCR5-agonists [34]. Indeed, a recent study performed in mice revealed that ECL1 and ECL2 induced stronger responses compared to the N-terminus, thus suggesting that different epitopes of CCR5 could trigger different receptor trafficking [38]. Of note, murine antibodies directed to ECL1 of CCR5 “in vivo” induced CCR5 internalization and re-expression following a very slow kinetics, similar to that observed in humans [39].

There are many published pieces evidence indicating that β-arrestins and MAPKs generally regulate downstream signaling pathways of different type of receptors and their signal complex could be relevant in the cross-communication integrating nodes [15]. However, the effect of the ERK/β-arrestin interaction on CCR5 receptor signaling pathway, upon stimulation with CCR5 antibodies, is not fully understood.

As published data have demonstrated that protein kinases inhibitors are able to block the interaction between β-arrestins and ERKs, here we demonstrate that the staurosporine (pan-kinases inhibitor) statistically significantly reduces the internalization with consequent cytoplasmic accumulation of CCR5 in a ligand-independent manner (Figure 1). Moreover, the data obtained in the immunoprecipitation assay conclusively proved that the agonist binding with CCR5 addresses the internalized receptor into early endosomes directly and the inhibition of protein kinases cascade deeply alters the protein–protein interaction among the CCR5 signalosome components, as demonstrated by decrease association with Rab5, an early endosomes marker. That suggests that the early temporal association of the internalized CCR5 with the endogenous ERK1 and β-arrestin2 requires a functional phosphorylation signal between the proteins involved in the CCR5 signalosome (Figure 2). Supporting these results, we have recently published that the absence of functional ERK1 protein, either by chemical inhibition or by using a dominant negative mutant, induces a reduction of both cytosolic CCR5 and β-arrestin1/2 accumulation in the presence of CCR5 Abs [11].

In addition, it was recently observed that the binding of natural ligands to CCR5 triggers the phosphorylation of four distinct C-terminal serine residues mediated by GPCR kinase (GRK) and protein kinase C (PKC) [17]. In particular, following ligand stimulation of CCR5, Serine 337 is solely phosphorylated by a PKC-mediated mechanism, while GRKs phosphorylate Serine 349 [12]. The inhibition of the phosphorylation cascade shows a relevant alteration on regulation of the core components of the CCR5 signaling pathway activation after exposure to anti CCR5 Abs. Moreover, inhibition of protein kinase activity results in a significant reduction of all major CCR5 signalosome players (Figure 3).

Of note, it is well known that, following receptor endocytosis mediated by stimulation with RANTES and its analogs, CCR5 accumulates in perinuclear recycling endosomes and return back unphosphorylated to the plasma membrane with a short term kinetics. In addition, CCR5 does not significantly colocalize with late endosomal and lysosomal markers [17]. Moreover, Bönsch and collaborators have published the different ability of two RANTES analogs (PSC and 5P14) to form durable complexes among CCR5 and β-arrestin1, even if the experiments have been performed at short time only (50 min) [40]. On the contrary, we have recently published the capability of CCR5 Abs to induce a switch from class A trafficking pattern, usually involved with natural ligands, such as RANTES, to a long lasting class B types [10].

Finally, the data on the interaction between CCR5 and Rab5, after silencing of either β-arrestin1 or β-arrestin2 in the presence of CCR5 Abs, highlighted that CCR5-Rab5 complex formation was evident only if β-arrestin2 was functional (Figure 4); this result suggests that β-arrestin2 is a crucial player to address the internalized receptor to early endosomes and not a scaffold protein to ERK1 only.

**Figure 5:**
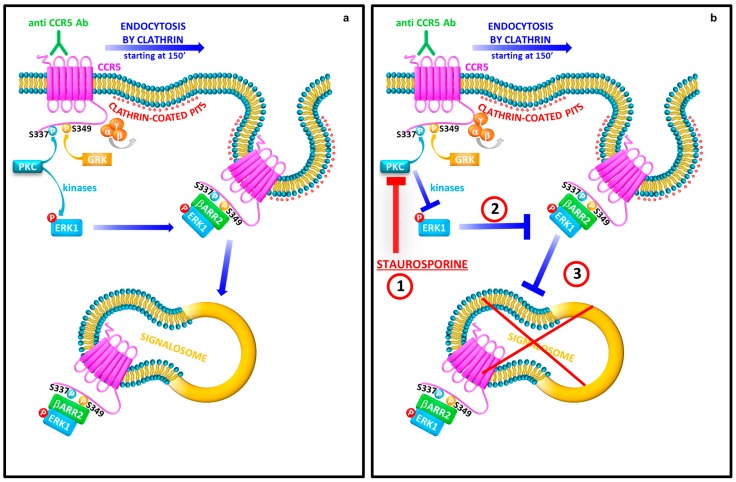
A mechanistic model of CCR5 signalosome formation mediated by CCR5 Ab Pos. CCR5 Ab Pos triggers the receptor phosphorylation GRK-mediated with the recruitment of β-arrestin2. (**a**) Remarkably, β-arrestin2 accumulates in complexes in association with the activated CCR5 and triggers the activation of ERK1, with consequent retention, into the cytosol (150 min); (**b**) the block of the protein kinases phosphorylation mediated by staurosporine treatment (1), which in turn has an effect on both CCR5 and ERK1 (2), resulting in the abolition of the accumulation of CCR5 into the early endosome after binding with CCR5 Ab Pos (3).

To summarize, upon stimulation with anti CCR5 antibodies, CCR5 is usually sequestered into complexes together with β-arrestin2 and ERK1, and driven to early endosomes. These events determine the formation of a stable CCR5 signalosome (150 min) (Figure 5, panel A).

Interestingly, the block of the protein kinases phosphorylation mediated by staurosporine treatment, that has an effect on both CCR5 and ERK1 proteins, abrogates the capture of the CCR5 signalosome into the early endosomes (Figure 5, panel B); this suggests the relevant role of phosphorylation events to form steady receptor–β-arrestin2 complexes, mediated by an ubiquitination of β-arrestin2 and a durable phosphorylation of ERK1, which are concentrated into the early endosomes.

Understanding the mechanisms for CCR5 signaling pathway is relevant to understand many aspects of cell and cancer biology as well as immunology, considering the involvement of this receptor not only in HIV-1 infection but also in many inflammatory disorders, including rheumatoid arthritis, multiple sclerosis [41] and tumorigenesis [42,43]. Furthermore, these findings could be used for effectively targeting signaling pathways using an antibodies-based therapy.
